# Gut microbiome-modulated dietary strategies in EAE and multiple sclerosis

**DOI:** 10.3389/fnut.2023.1146748

**Published:** 2023-03-29

**Authors:** Kristina Hoffman, William J. Doyle, Sean M. Schumacher, Javier Ochoa-Repáraz

**Affiliations:** Department of Biological Sciences, Boise State University, Boise, ID, United States

**Keywords:** diet, gut dysbiosis, multiple sclerosis, EAE, dietary factors, dietary interventions, gut microbiome

## Abstract

Over the last few decades, the incidence of multiple sclerosis has increased as society’s dietary habits have switched from a whole foods approach to a high fat, high salt, low dietary fiber, and processed food diet, termed the “Western diet.” Environmental factors, such as diet, could play a role in the pathogenesis of multiple sclerosis due to gut microbiota alterations, gut barrier leakage, and subsequent intestinal inflammation that could lead to exacerbated neuroinflammation. This mini-review explores the gut microbiome alterations of various dietary strategies that improve upon the “Western diet” as promising alternatives and targets to current multiple sclerosis treatments. We also provide evidence that gut microbiome modulation through diet can improve or exacerbate clinical symptoms of multiple sclerosis, highlighting the importance of including gut microbiome analyses in future studies of diet and disease.

## 1. Introduction

Multiple sclerosis (MS) is an autoimmune and neurodegenerative disease that affects the central nervous system (CNS). The estimated number of people with MS in 2020 was 2.8 million worldwide, with a global prevalence of 35.9 per 100,000 people (1). At the initial stages of the disease, T- and B-cell lymphocytes infiltrate into the parenchyma of the CNS, creating inflammatory and demyelination lesions mostly in the white matter but also in the gray matter. As the disease progresses, resident microglia and macrophages play an increasing role in the demyelination process (2). MS symptoms are highly variable and include visual interference, muscle weakness, and fatigue, which ultimately can lead to paralysis. MS disease onset and progression mechanisms are still under investigation due to many confounding factors, including genetics and environmental factors, such as viral infections (specifically Epstein Barr Virus), vitamin D deficiency, and diet. The incidence of MS has increased over the last few decades as people’s dietary habits have changed from a whole-food lifestyle to the “Western diet,” where people consume more processed foods with low dietary fiber, high fat, and high salt (3). The “Western diet” alteration of the gut microbiome is thought to play a role in the pathogenesis of MS and other autoimmune diseases (3). Intestinal inflammation through gut microbiota alterations could lead to gut barrier leakage and systemic inflammation (4). This intestinal disruption, associated with blood-brain barrier disruption, could lead to enhanced neuroinflammation (5, 6). Advancing our understanding of gut microbiome-modulated dietary strategies will contribute to the pathophysiology of MS. In this mini-review, we discuss the gut microbiome alterations with current dietary strategies that are not as widely studied in MS.

## 2. Gut dysbiosis and its role in EAE and MS

Gut microbes are closely co-evolved microbial partners interacting with the host and other microbial commensals. Gut microbes’ physiology and metabolism are exposed to environmental changes generated by the host’s genetics, immune system, and physiology (7), such as temperature, pH, oxygen, and others, and external modulators, such as diet and drugs. Alteration of this complex system by dietary factors could lead to inflammation and potential disease in gut dysbiosis. In this scenario, dietary factors could lead to dysbiosis, driving intestinal pro-inflammatory responses and subsequently leading to disease or exacerbation of the disease. Gut dysbiosis should then be considered when approaching auto-inflammatory conditions (8). Gut dysbiosis in the context of MS is supported by clinical evidence that indicates significant alterations in the microbiome composition of fecal content isolated from MS patients when compared with healthy individuals, as we recently reviewed (9, 10). Studying the gut microbial communities and their interactions is a complex task, as most taxa are not culturable in the lab. These technical limitations require the use of *in vivo* experimental models and sample collection studies based on genetic identification. A widely used animal model to study MS is experimental autoimmune encephalomyelitis (EAE). Despite its limitations, EAE is the most commonly used animal model for the study of the disease (11). EAE can be induced in multiple non-rodent and rodent species actively by injecting myelin oligodendrocyte glycoprotein (MOG) or other myelin self-antigens followed by pertussis toxin and passively by the adoptive transfer of encephalitogenic T-cells (12). EAE induction triggers an autoimmune response resulting in the proliferation and activation of pathogenic immune cells that cross the blood-brain barrier (BBB) and their infiltration into the CNS, leading to myelin sheath degradation by mechanisms that still need to be completely elucidated (13). EAE experiments have provided evidence that CNS inflammatory demyelination is linked to the gut microbiome by showing how antibiotic treatments impact disease onset and severity (14–18). Studies in germ-free (GF) mice showed that the lack of microbes results in reduced EAE severity (19, 20) and that the monocolonization of Th17-driving gut bacteria, such as segmented filamentous bacterium, restores the disease’s severity (19, 20). In addition, the reconstitution of GF mice with MS patient fecal content exacerbated EAE (21, 22). Creating detailed human microbiota profiles is a necessary step in translating the findings of EAE models (23).

Significant changes in gut microbiome composition have also been observed in both EAE models and MS clinical studies vs. healthy controls (HC) [for a discussion, see our latest reviews (9, 10)]. We recently summarized these studies in a different work (9). For the scope of this mini-review, we will discuss a few notable microbe alterations across EAE and MS studies to provide a base for dietary strategy comparison in the following sections and Table 1. In EAE studies, significant reductions in the relative abundances of microbes associated with anti-inflammatory responses were observed, including *Lactobacillaceae* (24, 25) and *Bacteroidaceae* (26). Inversely, there were increases in the abundances of *Clostridiaceae* (24, 25) and *Akkermansia* (24, 26). In MS vs. healthy control (HC) comparison studies, there were decreases in anti-inflammatory microbes *Bacteroidaceae* (27–30), and increases in pro-inflammatory microbes *Methanobrevibacter* (28, 31), *Acinetobacter* (21, 27, 32), and *Akkermansia* (21, 22, 30–32). Targeting specific microbes, their metabolites, or the pathways they play a role in for protection with dietary alterations is a potential novel avenue for treatment. For example, current research into the ratio of *Firmicutes* to *Bacteroidetes* (F/B) has shown it as a biomarker for dysbiosis and a potential biomarker of disease (33–35). Changes to this ratio are of high interest due to both bacterial phyla composing about 90% of the gut microbiome (35). High F/B has been associated with obesity, while low F/B with inflammatory bowel disease. However, there have been conflicting results, and many confounding factors are not considered, such as age, environmental conditions, and diet (33–35). In the following sections, we will briefly discuss various current dietary strategies and their impact on the gut microbiome in MS and EAE.

**TABLE 1:** Dietary alterations of the gut microbiome in EAE and MS.

Dietary strategies	Alterations of gut microbiome	Form of disease and impact	References**
No dietary alterations	EAE*: ↑ *Clostridiaceae* (24, 25) and *Akkermansia* (24, 26) ↓ *Lactobacillaceae* (24, 25) and *Bacteroidaceae* (26) MS*: ↑ *Methanobrevibacter* (28, 31), *Acinetobacter* (21, 27, 32), and *Akkermansia* (21, 22, 30–32) ↓ *Bacteroidaceae* (27–30)	EAE and MS disease alteration of gut microbiome to compare to dietary alterations	(22–32)
Vitamin D	MS: ↑ *Akkermansia*, *Faecalibacterium*, and *Coprococcus* (30)	MS: conflicting studies on the link of vitamin D and pathogenesis	(30)
Dietary fiber	EAE: ↑ *Ruminococcaceae*, *Helicobacteraceae* and *Enterococcaceae* (3) and *Lactobacillaceae* (52) ↓ *Sutterellaceae*, *Lactobacillaceae* and *Coriobacteriaceae* (3)	EAE: normal diet 55% disease incidence vs. cellulose rich diet 23%, and cellulose rich diet delayed neurological symptoms by about 1 week	(3, 52)
Mediterranean diet	EAE: no gut microbiome analysis, measured bacterial LPS levels MS: ↑ *Enterobacteriaceae*, *Akkermansia* and *Blautia* (80) ↓ *Faecalibacterium*, *Prevotella*, and *Parabacteroides* (80) Lower ratio *Firmicutes* to *Bacteroidetes* (80)	EAE: reduced bacterial endotoxins and reduced oxidative stress MS: antioxidant and anti-inflammatory	(80)
Isoflavone diet	EAE: ↑ *Adlercreutzia* and *Parabacteroides* (3, 82) ↓ *Akkermansia* (3, 82)	EAE: increased bacteria are deficient in MS and decreased bacteria associated with MS	(3, 82)
High vegetable/Low protein	MS high vegetable/low protein: ↑ *Lachnospiraceae*, *Coprococcus*, *Ruminococcus*, a sequence of an unclassified *Lachnospiraceae* strain, *Roseburia*, and a *Hungatella*-related unknown *Lachnospiraceae* (34) MS “Western diet”: ↑ *Euryarchaeota* (83)	MS: samples of patients on different diets were taken after 12 months, no baseline samples for comparison	(34, 83)
Intermittent fasting	EAE: ↑ *Bacteroidaceae*, *Lactobacillaceae* and *Prevotellaceae* (85) ↓ *Bifidobacterium* (85) MS: pilot study with similar alterations to EAE (85)	EAE: delayed onset and reduced severity MS: intermittent fasting partly linked to altered gut microbiome beneficial effects	(85)

*The prevalent microbes across studies shown.

**References are organized in order of dietary strategies.

## 3. Dietary factors in EAE and MS

In this section, we discuss the dietary factors shown to regulate experimental disease in the EAE model and potential relevance to human disease. Common components of daily dietary habits, such as salt and zinc, are known to regulate EAE severity. The effects of high concentrations of sodium chloride seem to be triggered by the induction of Th17 cell-mediated proinflammation (36–38). Dietary sodium was also shown to exacerbate EAE (39). By contrast, when combined with aspartate, the treatment with zinc and salts reduced disease severity (40). Prophylactic treatment with zinc dissolved in drinking water also reduces EAE clinical scores by modulating Treg/Th17 balances, promoting immunosuppression (41). Below, we summarize the most widely studied dietary factors as modulators of EAE. These and other dietary factors capable of regulating EAE severity were revised extensively by van den Hoogen et al. (42).

### 3.1. Fibers and derived metabolites

The supplementation of diet with fibers is proposed as a supplement strategy for MS patients due to their reduced abundance in the “western diet” (43). Dietary fiber can be either soluble or insoluble and fermentable or non-fermentable complex carbohydrates. Fermentable fiber microbial end products, short chain fatty acids (SCFAs), have evidence supporting protective and therapeutic properties in autoimmune diseases such as MS (3). SCFAs promote a healthy gut and provide systemic effects that could result in beneficial effects against neuroinflammation. As we recently reviewed, an increasing number of studies indicated the potential of triggering SCFA-specific responses in the context of inflammation (9). SCFAs promote anti-inflammatory pathways that regulate the balance between Th17 and Treg responses. Colonic bacteria that produce SCFAs are reduced in fecal samples from MS individuals compared to HC samples (44). Circulating levels of SCFAs appear to be reduced in MS individuals, and they are protective in the EAE model (45), promoting axonal remyelination (46–50). SCFAs produced by colonic bacteria have also been shown to be key to maintaining the integrity of the BBB (51).

Non-fermentable fiber is a common vegetarian diet component and has yet to be as thoroughly investigated due to poor breakdown *via* gut microbes. In a recent clinical study, non-fermentable fiber dietary cellulose has been shown to increase amounts of long-chain fatty acids and alter gut microbiota’s composition, promoting beneficial effects in MS (3). Experimentally, in animals administered with normal diet, the EAE disease incidence was 55% compared to the 23% incidence observed in animals fed with a cellulose-rich diet (3). Neurological symptoms were significantly delayed about 1 week in the cellulose-rich diet EAE group; however, there was no significant difference in disease severity (3). Despite the reduction in SCFA-producing microbes and increase in long-chain fatty acids, the cellulose-rich diet demonstrated beneficial effects (3). The fermentable dietary fiber guar gum reported increased SCFAs and significantly delayed EAE (43). A notable mention that might benefit from additional gut microbiome studies is pomegranate peel extract which increased *Lactobacillaceae* and reduced the severity of EAE (52). Pomegranate peel extract contains dietary fiber, polyphenols, vitamins, and minerals (53). These studies support dietary fibers as potent modulators of EAE and potential protective or therapeutic supplements for MS patients. A more detailed discussion of dietary fibers and their impact on the gut microbiome was recently reviewed by others (54).

Experimental autoimmune encephalomyelitis studies also revealed the protective effects of polyunsaturated fatty acids (FAs) against neuroinflammation, such as omega-6 fatty acid γ-linolenic acid in SJL mice (55), and oil-containing linolenic acids in Lewis rats (56), as well as docosahexaenoic acid (57) and eicosapentaenoic acid (58, 59) in C57BL/6 mice. Interestingly, the protective effects of omega-6 fatty acid γ-linolenic acid in the EAE model contradict previous findings of omega-6 as a pro-inflammatory factor. The notion of omega-6 FAs as precursors of pro- or anti-inflammatory mediators have been discussed by others (60). The form of omega-6 and their interactions with omega-3 FAs might be relevant to the nature of the immunological responses triggered (60). Diet supplementation with omega-3 resulted in moderated beneficial effects in a clinical study (61). These and other dietary factors that regulate EAE severity were already revised (42).

### 3.2. Vitamin supplementation

Vitamin D supplements have been proposed for use combined with disease-modifying therapies against MS. Vitamin D is fat-soluble, naturally present in foods, can be supplemented, and is stimulated from skin exposure to sunlight. Vitamin D plays a role in increasing intestinal absorption and retention of relevant nutrients such as calcium. Vitamin D deficiency is a common worldwide issue, even with the supplementation previously stated. Due to the distribution of MS being more prevalent where there is less sun exposure, vitamin D deficiency is thought to play a role in the pathogenesis of the disease (62). In an exploratory clinical study, MS patients were evaluated for alterations in the gut microbiota with vitamin D. MS patients treated with vitamin D had increased *Akkermansia*, *Faecalibacterium*, and *Coprococcus* vs. untreated MS (30). These microbes tend to be associated with pro-inflammatory properties. In other clinical studies measuring vitamin D levels, there is no consistent link between vitamin D supplementation and reduction or protection of MS (63). The combination of vitamin D and MOG protected against EAE (64). In addition, the administration of vitamin D3 also protected against the experimental disease (65). Another study showed that the protection induced by vitamin D treatment depends on the age of the animals and suggests that early administration of the dietary factor could be preferred (66). Interestingly, the requirement for vitamin D and vitamin D receptors for EAE induction has also been documented (67). A more detailed discussion on the role of vitamin D in regulating neuroinflammation has been recently published (68).

Vitamin A metabolites such as retinoid acid are protective against EAE by regulating the Treg-Th17 axis and Treg function (69–71). The immunomodulatory effects of retinoic acid have been demonstrated in other autoinflammatory models (72, 73).

The effects of vitamin E have also been explored in murine models of EAE through the administration of tocopherol or its derivatives (74, 75). In addition to reductions in EAE clinical scores, vitamin E (and vitamin D3) was also associated with increased remyelination in a toxic rat model of demyelination by injection of ethidium bromide (76).

## 4. Dietary interventions in EAE and MS

In the following paragraphs, we discuss studies that applied dietary interventions in MS patients and supporting pre-clinical evidence using EAE models. For example, the marmoset model EAE was used to address the potential beneficial effects of a diet enriched in fruit juices, carrot juice, yogurt, yeast flakes, vitamin D, among other components, and reduced ground powder. The administration of the diet selected a Bifidobacteria-enriched microbiome and reduced pro-inflammatory responses, inducing neuroprotection (77).

### 4.1. Mediterranean diet

The Mediterranean diet has been proposed to benefit cognitive health (78) and even be neuroprotective (79). The diet is associated with a lower ratio of *Firmicutes* to *Bacteroidetes* and promotes microbial diversity, which is important for gut homeostasis (80). Olive oil is one of the primary ingredients in the Mediterranean diet, in addition to large amounts of fruits, vegetables, whole grains, seafood, nuts, and legumes. Extra-virgin olive oil has previously been shown to have antioxidant and anti-inflammatory properties due to mono-unsaturated fats and phenols (81). Although a gut microbiome analysis was not performed in this extra-virgin olive oil EAE study, it is notable that the investigators detected increased circulating levels of lipopolysaccharide, Gram-negative bacterial endotoxin, in control EAE mice (81). Lipopolysaccharide levels were reduced in animals fed extra-virgin olive oil, suggesting dysbiosis and increased intestinal barrier disruption. Olive oil-fed EAE mice also showed reduced oxidative stress compared to controls (81).

Further supporting evidence is needed to demonstrate how diet modification can impact the gut microbiome and subsequently have a neuroprotective effect. A clinical study evaluated the impact of a Mediterranean diet on the microbiome of MS patients in a Spanish cohort of MS vs. HC patients (80). The study showed significant differences in the microbiota composition of MS and HC samples. Although the results indicated potential beneficial changes on the microbiota composition when exposed to the Mediterranean diet, the samples obtained from the MS cohort showed increased *Enterobacteriaceae*, a bacterial family that contains several potential pathogens (80).

### 4.2. Isoflavone diet

An isoflavone diet is characterized as a legume-based diet. Dietary legumes are rich in isoflavones and can include leaves, stems, and the fruit or seeds of certain plants, such as beans and peas. Isoflavones are phytoestrogens. Humans do not have the enzymes to break down isoflavones, so we rely on gut microbes for their catabolism and use. Isoflavone-metabolizing bacteria have previously been shown to be reduced in MS and are known for their antioxidant and anti-inflammatory properties for other diseases such as cancer (82). A recent EAE study investigated the effects of diets with and without isoflavones on the composition of the gut microbiome (82). The study found that the isoflavone-rich diet increased the isoflavone-metabolizing microbes *Adlercreutzia* and *Parabacteroides* in EAE mice. These bacteria are reduced in MS patients. By contrast, the administration of the diet without isoflavone to EAE mice increased the relative abundances of *Akkermansia* (82). These results suggest an isoflavone diet may have protective and therapeutic effects in neuroinflammatory diseases.

### 4.3. High vegetable/low protein diet

In line with previous diets, a diet high in vegetables and low in protein has been shown to improve MS clinical symptoms (83). A clinical pilot study was performed with MS patients comparing a high vegetable/low protein diet with the “Western diet” after a minimum of 12 months on the diet. Increased *Lachnospiraceae*, *Coprococcus*, *Ruminococcus*, a sequence of an unclassified *Lachnospiraceae* strain, *Roseburia*, and a *Hungatella*-related unknown *Lachnospiraceae* along with reduced disease severity and relapse were found in MS high vegetable/low protein diet patients (83). *Lachnospiraceae* has been associated with anti-inflammatory properties (83). In the MS “Western diet” patients, there was a significant increase in *Euryarchaeota* (83). For this pilot study, it is important to note they did not take baseline samples before the start of the diets for analysis, so the comparison for alteration of the gut microbiomes is strictly between MS patient diets at the end of a minimum of 12 months (83).

### 4.4. Reduced carbohydrate consumption and intermittent fasting

Ketogenic diets drastically reduce the intake of carbohydrates, inducing ketosis and driving the production of ketone bodies, an alternative energy source for the brain (84). Ketone bodies are believed to promote myelin regeneration, a possible target for therapeutics and protection in MS (84). Intermittent fasting also produces ketone bodies by inducing ketosis (84). Both may activate autophagy pathways that ameliorate disease by renewing and mobilizing non-nuclear and cytoplasmic macromolecules in cells (84). EAE studies have shown the beneficial effects of intermittent fasting; however, clinical studies are limited (84). Bahr et al.’s study is the first clinical study investigating ketogenic and intermittent fasting effects, which they found may modulate MS. However, clinical evidence was lacking. No gut microbiome analysis was performed. A recent EAE intermittent fasting study showed delayed onset and reduced severity of EAE (85). In the intermittent fasting EAE, there was an increase in anti-inflammatory microbes *Bacteroidaceae*, *Lactobacillaceae*, and *Prevotellaceae*, and a decrease in pro-inflammatory microbes *Bifidobacterium* (85). Cignarella et al. also performed a pilot clinical trial and found gut microbiome alterations resembling protective microbe alterations in their EAE study. They found intermittent fasting to have protective and therapeutic effects linked partly to an altered gut microbiome in EAE and MS.

## 5. Discussion

The studies summarized in this mini-review highlight the importance of future investigation into dietary interventions, specific microbes associated with dietary interventions, their interactions, and the roles they play in biological processes within EAE and MS. This mini-review covered various current and popular dietary strategies; however, the gut microbiota can produce thousands of metabolites and plays a role in many biological processes that need to be further investigated (80). The EAE model has extended our understanding of the multifactorial interactions between the gut microbiota, diet, and host. Less is known about the effects of diet on the MS cohort. The mini-review also highlights the need for more and larger clinical studies evaluating dietary strategies in MS patients, including the impact on the gut microbiome. Nevertheless, the number of studies evaluating the impact of diet on the immune responses and signaling molecules in MS have increased of recent years (42, 86). Future studies that include analyses of gut microbiome modulation *via* dietary strategies are needed to investigate further the mechanisms for use as targets in the protection or treatment of MS.

## Author contributions

KH designed the outline of the manuscript. WD, SS, and JO-R helped with edits and revisions. All authors contributed to the article and approved the submitted version.
